# *Actinoplanes teichomyceticus *ATCC 31121 as a cell factory for producing teicoplanin

**DOI:** 10.1186/1475-2859-10-82

**Published:** 2011-10-18

**Authors:** Carlo Taurino, Luca Frattini, Giorgia Letizia Marcone, Luciano Gastaldo, Flavia Marinelli

**Affiliations:** 1 Farmhispania S.A., Montmelò, Barcelona and Rolabo Outsourcing S.L, Zaragoza, Spain; 2 Dipartimento di Biotecnologie e Scienze della Vita, Università degli Studi dell'Insubria, Varese, and The Protein Factory, Centro Interuniversitario di Ricerca in Biotecnologie Proteiche, Politecnico di Milano and Università degli Studi dell'Insubria, Italy

## Abstract

**Background:**

Teicoplanin is a glycopeptide antibiotic used clinically in Europe and in Japan for the treatment of multi-resistant Gram-positive infections. It is produced by fermenting *Actinoplanes teichomyceticus*. The pharmaceutically active principle is teicoplanin A_2_, a complex of compounds designated T-A_2-1_-A_2-5 _differing in the length and branching of the fatty acid moiety linked to the glucosamine residue on the heptapeptide scaffold. According to European and Japanese Pharmacopoeia, components of the drug must be reproduced in fixed amounts to be authorized for clinical use.

**Results:**

We report our studies on optimizing the fermentation process to produce teicoplanin A_2 _in *A. teichomyceticus *ATCC 31121. Robustness of the process was assessed on scales from a miniaturized deep-well microtiter system to flasks and 3-L bioreactor fermenters. The production of individual factors T-A_2-1_-A_2-5 _was modulated by adding suitable precursors to the cultivation medium. Specific production of T-A_2-1_, characterized by a linear C10:1 acyl moiety, is enhanced by adding methyl linoleate, trilinoleate, and crude oils such as corn and cottonseed oils. Accumulation of T-A_2-3_, characterized by a linear C10:0 acyl chain, is stimulated by adding methyl oleate, trioleate, and oils such as olive and lard oils. Percentages of T-A_2-2_, T-A_2-4_, and, T-A_2-5 _bearing the iso-C10:0, anteiso-C11:0, and iso-C11:0 acyl moieties, respectively, are significantly increased by adding precursor amino acids L-valine, L-isoleucine, and L-leucine. Along with the stimulatory effect on specific complex components, fatty acid esters, oils, and amino acids (with the exception of L-valine) inhibit total antibiotic productivity overall. By adding industrial oils to medium containing L-valine the total production is comparable, giving unusual complex compositions.

**Conclusions:**

Since the cost and the quality of teicoplanin production depend mainly on the fermentation process, we developed a robust and scalable fermentation process by using an industrial medium in which a complex composition can be modulated by the combined addition of suitable precursors. This work was performed in the wild-type strain ATCC 31121, which has a clear genetic background. This is important for starting a rational improvement program and also helps to better control teicoplanin production during process and strain development.

## Background

**Figure 1:**
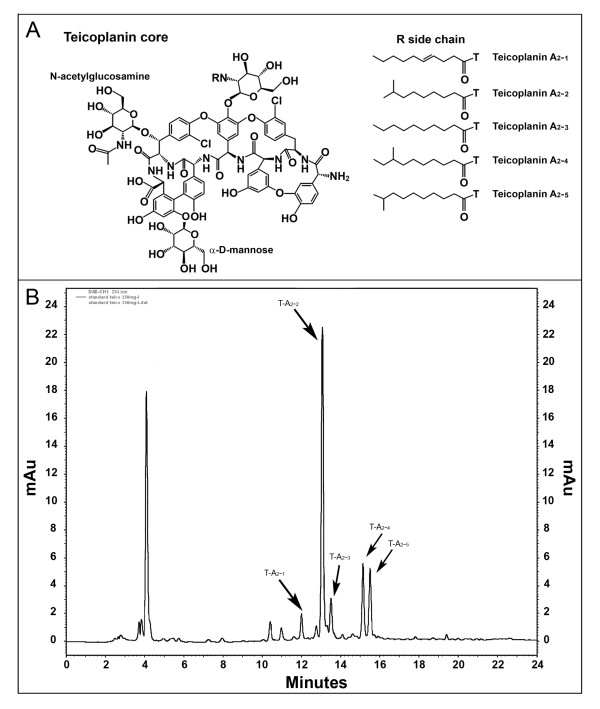
**Teicoplanin A_2 _(T-A_2_) chemical structure and Targocid complex**. In **A**, the chemical structure of teicoplanin core shows that the different components T-A_2-1_, T-A_2-2_, T-A_2-3_, T-A_2-4_, and T-A _2-5 _vary in the acyl chain linked to the glucosamine residue on the heptapeptide scaffold. In **B**, a pure sample of teicoplanin (Targocid, Sanofi-Aventis) was analyzed by the HPLC method described in the Methods. In these conditions, T-A_2-1_, T-A_2-2_, T-A_2-3_, T-A_2-4_, and T-A _2-5 _represent 6, 58.3, 7.3, 14.4, and 14% of the total T-A_2_.

Glycopeptide antibiotics such as vancomycin and teicoplanin are frequently used to treat life-threatening infections caused by multi-drug-resistant Gram-positive pathogens. They are linear heptaptides produced by nonribosomal peptide synthetase, further oxidatively linked among aromatic amino acids and decorated with chlorine atoms, glycosidic moieties, and (in the case of teicoplanin) lipid chains. Vancomycin, introduced into clinical practice in 1958, is produced from the actinomycete *Amycolatopsis orientalis*, whereas teicoplanin, used in hospitals in Europe and Japan since 1988 and 1998, respectively, is from *Actinoplanes teichomyceticus *[1-3]. Both compounds are known to inhibit the formation of the bacterial cell wall by binding to the N-acyl-D-alanyl-D-alanine terminal of lipid II, thereby terminating the subsequent transpeptidation and transglycosylation reactions in the late extracellular stages of peptidoglycan biosynthesis. Lipoglycopeptides such as teicoplanin and its derivatives are reported to be more effective than vancomycin against methicillin-resistant *Staphylococcus aureus*, as they carry an extra aliphatic acyl side chain on glucosamine at residue 4 (Figure 1A). Such a modification may enable lipoglycopeptides that are anchored on the lipid layer of the bacterial membrane to be close to cell wall precursors [1,2].

Although vancomycin and teicoplanin have been completely synthesized, the complexity of these natural products renders fermentation the only viable route for producing them pharmaceutically. Teicoplanin is produced by fermenting *A. teichomyceticus *ATCC 31121, as a complex of structurally related molecules differing in the length and branching of the fatty acid moiety linked to the glucosamine residue on the heptapeptide scaffold [4]. The common core of teicoplanin (Figure 1A) consists of seven aromatic amino acids, which, apart from the tyrosine residue at position 2, are nonproteinogenic amino acids: three *p*-hydroxyphenylglycine residues at positions 1, 4, and 5; two dihdroxyphenylglycine at positions 3 and 7; and one β-hydroxytyrosine at position 6. Three ether bonds link amino acids at positions 1-3, 2-4, and 4-6, and one C-C link between amino acids 5-7 joins aryl groups. The tyrosine and the β-hydroxytyrosine residues at positions 2 and 6 contain a chlorine atom. Three sugar moieties are attached to the aryl groups: a α-D-mannose at amino acid 7, a N-acetyl-β-D-glucosamine at amino acid 6, and a N-fatty acyl-β-D-glucosamine at position 4. Enzymes involved in synthesizing the precursor aromatic nonproteinogenic amino acids, assembling them in the heptapetide precursor, and cross-linking, halogenating, and adding sugars to them are encoded by genes clustered on the *A. teichomyceticus *chromosome [5,6]. The acyl chains linked to glucosamine at position 4 are linear or branched nine-, ten-, eleven-, twelve-carbon acids thought to be derived from ß-oxidation of longer fatty acids contained in the membrane pool of *A. teichomyceticus *[7].

The pharmaceutically active principle produced by fermentation is teicoplanin A_2 _(T-A_2_), a complex of five related compounds designated teicoplanin A_2-1_-A_2-5_, characterized by five different linear or branched ten- or eleven-carbon fatty acids (Figure 1A), which accounts for 89 to 95% of the total teicoplanin complex. A sixth, more polar active component, teicoplanin A_3_, which is not found in fermentation broth, is always present in crude or purified extracts; it is a hydrolytic product of T-A_2 _[8]. Especially according to the European Pharmacopoeia, which is much more restrictive than the Japanese guidelines [9], the six subcomponents should be present in the active pharmaceutical ingredient in rigorously fixed proportions. Although it is unlikely that small variations in the composition of active fractions significantly affect the biological activity and/or safety and efficacy of the drug *in vivo*, nowadays the responsible authorities do not permit the Pharmacopoeia specifications of complex composition to be different from the one registered for the innovator drug Targocid (Figure 1B).

Since the cost and the quality of teicoplanin depend mainly on the fermentation process, we investigated whether *Actinoplanes teichomyceticus *ATCC 31121 could be used as a cell factory for producing the drug. Particular attention was given to modulating the complex composition by adding fatty acid precursors or their amino acid precursors to the fermentation process, due to the importance for the aforementioned manufacturing guidelines.

## Results

### Single-factor optimization of teicoplanin production medium

Four complex industrial media previously described in the original patent and in the more recent literature on producing teicoplanin by *Actinoplanes teichomyceticus *were used to assay T-A_2 _production in the ATCC 31121 strain: E1 [10], TE/20 [7], Medium 1 [11], and Medium 2 [12] were selected since they differ mainly in carbon and nitrogen sources and salt content. Briefly, *A. teichomyceticus *grew well in these media (protocols to prepare reproducible cultures are described in the Methods) but produced only from 0 to 25 mg/L T-A_2_. The highest productivity was obtained in TE/20 (containing glucose at 20 g/L and maltose at 20 g/L as carbon sources, cottonseed meal at 15 g/L, and yeast extract at 6 g/L as nitrogen sources, and CaCO_3 _at 6 g/L as the only added salt), which was then selected as our starting point for further optimization by quantitatively/qualitatively changing its components in single-factor experiments.

**Figure 2:**
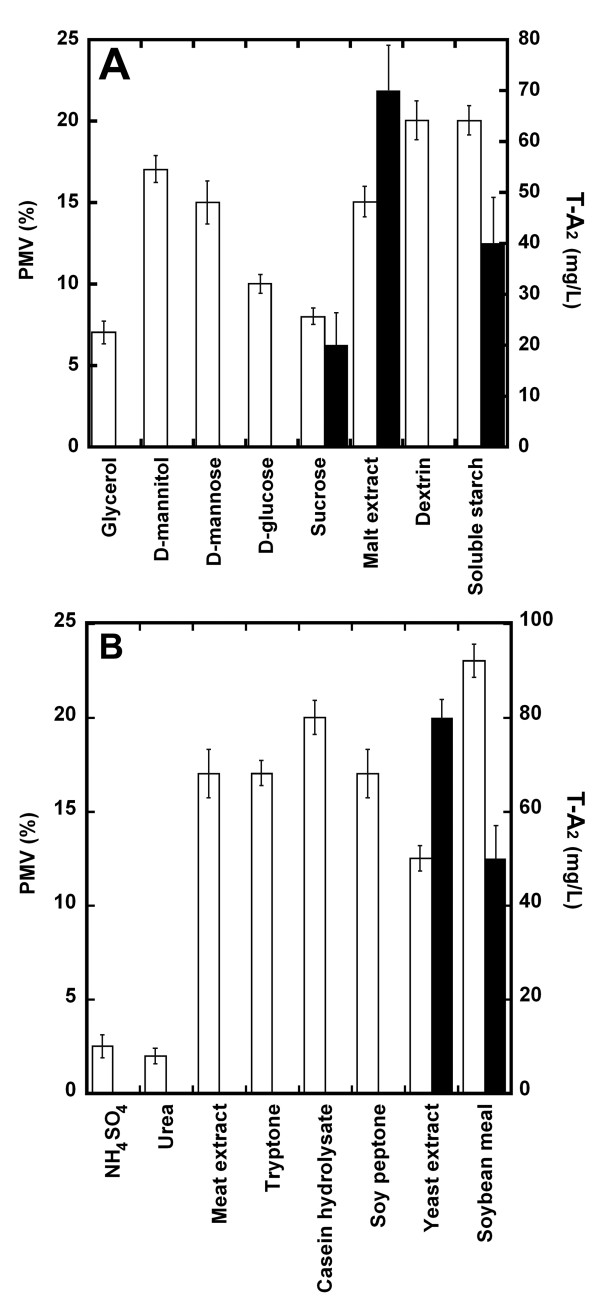
**Optimization of teicoplanin production medium**. Effect of different carbon (**A**) and nitrogen (**B**) sources on growth (PMV, empty bars) and T-A_2 _production (mg/L, filled bars) by *A. teichomyceticus *ATCC 31121. Carbon sources were tested at 20 g/L replacing maltose in TE/20. Nitrogen sources were tested at 15 g/L replacing cottonseed meal in TE/20.

Various carbon sources, including glycerol, D-mannitol, D-mannose, D-glucose, sucrose, malt extract, dextrin, and soluble starch (all at the concentration of 20 g/L), were tested in TE/20 with regard to the growth of *A. teichomyceticus *(measured as Packed Mycelium Volume, PMV) and the production of T-A_2_, respectively. The results (Figure 2A) revealed that all these sugars sustained biomass production to different extents, but malt extract, an industrial component often used by the fermentation industry, was the most effective carbon source to support T-A_2 _production, which subsequently improved to 70 mg/L. Inorganic nitrogen sources (15 g/L) such as ammonium sulfate, simple organic sources such as urea, and protein complex hydrolysates such as meat extract, hydrolysate of casein, tryptone, soy peptone, yeast extract, and soybean meal were tested next, replacing cottonseed meal in TE/20, respectively. Figure 2B shows that soybean meal was the best nitrogen source for sustaining biomass production, whereas yeast extract was the best candidate for producing T-A_2_. Two industrially used derivatives from cottonseed meal, Pharmaflo and Pharmamedia (Pharmamedia Archer Daniels Midland Company, IL, USA), were also tested as possible alternatives to soybean meal, but they did not show significant improvement either in biomass or T-A_2 _production (data not shown) and were thus abandoned.

**Figure 3:**
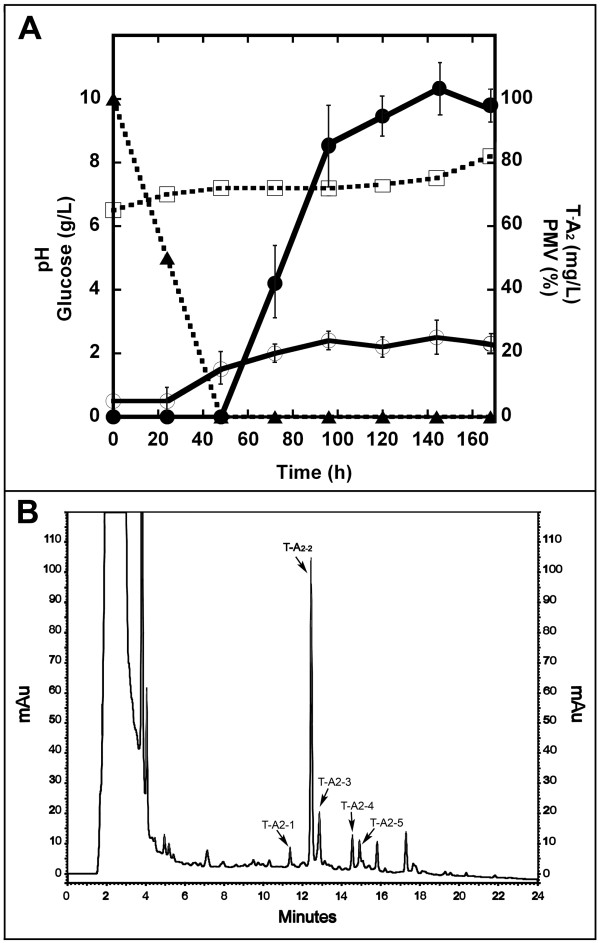
**Fermentation of *A. teichomyceticus *ATCC 31121 at flask scale in TM1 medium**. In (**A**), time courses of pH (□, dashed line), glucose (▲, dashed line), growth curve measured as PMV (○, solid line), and T-A_2 _production (●, solid line). In (**B**), HPLC profile of 144-hour sample showing the following complex composition: T-A_2-1_, T-A_2-2_, T-A_2-3_, T-A_2-4_, and T-A _2-5 _represent 7.3, 60.2, 13.1, 9.1, and 10.3% of the total T-A_2_.

In a following set of experiments (not shown), different concentrations of glucose (10, 20, 30 g/L) and malt extract (10, 20, 30 g/L) as carbon sources and soybean meal (5, 15, 20 g/L) and yeast extract (5, 15, 20 g/L) as nitrogen sources were combined and antibiotic production evaluated. The optimal concentration of malt extract was found to be 30 g/L in the presence of 10 g/L glucose, and a combination of soybean meal at 15 g/L and yeast extract at 5 g/L supported higher titers of T-A_2. _Under these conditions, 100 mg/L (σ = ± 14, 6 mg/L) T-A_2 _was produced at flask fermentation scale (Figure 3A). Finally, different concentrations of CaCO_3_, which was the only salt added to the initial TE/20 medium, were also tested. By removing CaCO_3 _or adding it at 2 g/L, T-A2 production was reduced by 70 and 40%, respectively. No changes in T-A_2 _production were achieved by adding 4 or 6 g/L CaCO_3_. Thus, the novel T-A_2 _production medium was determined to consist of 30 g/L malt extract, 10 g/L glucose, 15 g/L soybean meal, 5 g/L yeast extract, and 4 g/L CaCO_3_. This complex industrial medium was termed Teicoplanin Medium 1 (TM1).

In TM1 flask fermentation, onset of growth was observed after a lag phase of about 20 hours, which coincided with a slight increase in medium pH. A rapid decrease in free glucose concentration preceded the start of T-A_2 _production, which ensued at *ca*. 48 hours after inoculation and reached its maximum at 120-144 hours (Figure 3A). Under these conditions, the T-A_2 _complex showed the subcomponent composition reported in Figure 3B.

### Fermentation from a miniaturized system to bioreactor scale

*A. teichomyceticus *growth and T-A_2 _production were tested in commercially available, square, deep-well polypropylene microtiter plates (24-SDW MTPs) with sandwich covers (System Duetz [13]), consisting of 24 wells filled with 2.5 mL production medium each. We thought that deep-well plates could considerably reduce the work load and cost of the investigation while optimizing the medium and improving the strain. For growing filamentous microorganisms to produce antibiotics in industrially and not completely soluble rich media, the use of such miniaturized systems is often limited by insufficient aeration and poor mixing [13-15].

**Figure 4:**
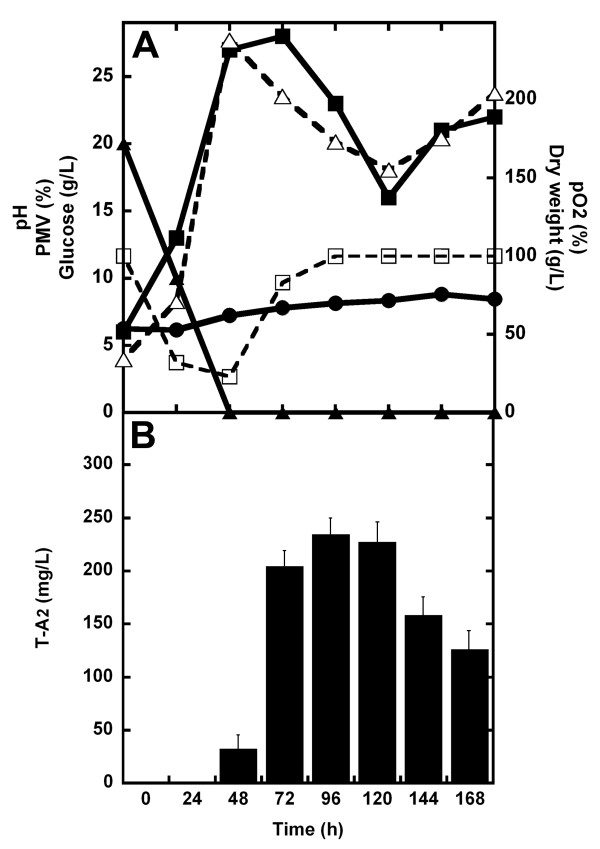
**Growth and teicoplanin production in 3-L batch fermentations of *A. teichomyceticus *ATCC 31121 in TM1**. The pH value was naturally self-regulated (*i.e*., it was not controlled by adding acid/base during the fermentation), whereas the pO_2 _was controlled over the 20% of saturation by adjusting agitation speed. In (**A)**, time courses of pH (●, solid line), pO_2 _(□, dashed line), glucose (▲, solid line), and growth curve measured as dry weight (Δ, dashed line) and PMV (■, solid line). In (**B**), production of T-A_2 _measured by HPLC analysis as mg/L (filled bars).

As described in the Additional file 1, the dissolved oxygen (DO) concentration is a crucial parameter in T-A_2 _production by *A. teichomyceticus*. The teicoplanin dependence on DO was indirectly demonstrated by increasing the volume of production medium (thus reducing the oxygen transfer rate) in 300 ml Erlenmeyer flaks: T-A_2 _titer decreased when 300 ml Erlenmeyer flasks were filled with increasing working volumes of TM1 (Additional file 1, Figure S1). The direct effect of DO (measured as % pO_2_) on T-A_2 _production was monitored at the bench reactor scale: comparison of Figure S2 (Additional file 1) and Figure 4 (see below) demonstrates the positive effect of controlling pO_2 _by varying the agitation speed. In uncontrolled, 3-L fermentations runs, where pO_2 _rapidly tended to zero in 25 hours, 25-30% less T-A_2 _was produced (Figure S2, Additional file 1) in comparison to those runs in which pO_2 _was maintained above 20% of saturation (Figure 4).

Notwithstanding this dependence on oxygen rate, T-A_2 _production was well reproducible in 24-SDW MTPs: the average productivity in the 24 wells at 120-144 hours was 100 mg/L (σ = ± 10 mg/L), which compared favorably with data obtained in flasks. The HPLC profile of T-A_2 _complex composition in 24-SDW MTPs overlapped with the one shown in Figure 3B. This good performance of the miniaturized System Duetz is likely due to the favorable volume (2.5 mL working volume in 12.5 mL total well volume), geometry (square-instead of classical round-bottom wells), and special covers, which allowed agitation on a rotatory shaker and avoided evaporation and cross-contamination [13-15].

The robustness of the teicoplanin fermentation process was also confirmed by scaling it up to fermenters. Figure 4 shows a typical time course of *A. teichomyceticus *fermentation in TM1 at a 3-L fermenter scale, where pO_2 _was maintained over 20% of saturation (see also Figure S2, Additional file 1). Growth curve monitored by dry weight indicate that *A. teichomyceticus *exponentially grew up to 48 hours after inoculation, then growth rate tended to diminished, and after 120 hours from the inoculation a secondary exponential growth phase is observed as previously shown by other authors [16]. Glucose consumption was completed in the first 48 hours and preceded T-A_2 _production, which reached its maximum at 96-120 hours after inoculation (volumetric productivity of 234 mg/L of culture with σ = ± 16 mg/L, specific productivity of 1.3 mg/g of biomass). pH tended to increase slowly after the first 24 hours of fermentation from 6, reaching 8 during the last phase of the process. Fermentations run by controlling pH at 7 by adding acid after 24-48 hours of growth did not improve T-A_2 _production (Figure S3, Additional file 1) and thus we did not introduce pH control in our reference fermentations. The HPLC profile of T-A_2 _composition at 120 hours of fermentation described in Figure 4 overlaps with that shown in Figure 3B.

### Modulation of teicoplanin complex composition

The production of individual T-A_2 _factors was modulated by adding suitable precursors to the industrial production medium TM1. Factors T-A_2-1 _and T-A_2-3 _are characterized by linear 4-n-decenoic (n-C10:1) and n-decanoic (n-C10:0) fatty acids, respectively, linked by an amide bond to the glucosamine residue (Figure 1). Previous data [7,17] showed that 4-n-decenoic and n-decanoic fatty acids in teicoplanin derive from β-oxidation of linoleic (n-C18:2) and oleic (n-C18:1) acids, respectively. We investigated the effect of adding these C-18 fatty acids as free acids, methyl esters, and triglycerides to TM1 on T-A_2 _production and finally of supplying them as main components in raw materials such as available crude oils. Adding free acids was ruled out immediately due to their toxicity even at low concentrations (0.5 g/L), which blocked *A. teichomyceticus *growth. Different concentrations of methyl esters of oleic acid and linoleic acids were added to the fermentation medium at the time of inoculation. As shown in Table 1, adding methyl linoleate or trilinoleate stimulated the production of T-A_2-1 _factor, which reached 20-30% of the total T-A_2 _complex, whereas methyl oleate and trioleate increased the relative T-A_2-3 _productivity to 30-50%. Indeed, a common feature of adding these esters was the dose-dependent reduction in T-A_2 _complex production as a whole. A concentration of methyl linoleate higher than 2 g/L abolished T-A_2 _production.

**Table 1:** Effect of biosynthesis precursors on teicoplanin production.

		Percentage composition				
		
Addition	Total T-A_2_	T-A_2-1_	T-A_2-2_	T-A_2-3_	T-A_2-4_	T-A_2-5_
None	100	7.3	60.2	13.1	9.1	10.3

Methyl linoleate						
0.5 g/L	31	3.2	49.7	16.9	17.1	13.1
1 g/L	28	10.6	49.3	17.0	13.0	10.1
2 g/L	17	29.0	31.0	18.8	10.0	11.2
4 g/L	0	0	0	0	0	0
6 g/L	0	0	0	0	0	0

Trilinoleate						
2 g/L	21	21.9	43.1	15.1	10.1	9.8

Methyl oleate						
0.5 g/L	38	2.8	56.2	16.3	13.1	11.6
1 g/L	17	2.2	46.2	20.3	19.3	12.0
2 g/L	12	4.0	40.2	25.7	14.9	15.2
4 g/L	10	7.0	31.6	30.7	16.7	14.0
6 g/L	9	5.8	29.9	31.1	17.7	15.5

Trioleate						
2 g/L	75	7.6	31.8	49.7	6.1	4.9

L-valine						
1 g/L	160	6.2	77	12.9	1, 9.	2
2 g/L	191	6.1	80	11.9	1	1
3 g/L	148	6.3	74	14.2	3	2, 5

L-isoleucine						
1 g/L	65	10	42	13	28	7
2 g/L	50	11.1	39.1	12.6	30	7.2
3 g/L	32	10	34.3	14	35.5	6, 2

L-leucine						
1 g/L	85	7	50	13	10	23
2 g/L	70	9	49	15	2	25
3 g/L	60	8.1	48	16.6	3.9	23.4

*A. teichomyceticus *ATCC 31121 was grown in TM1 medium added at the time of inoculum with different biosynthetic precursors. Samples were harvested at 144 hours and analyzed by HPLC as reported in the Methods. Total T-A_2 _complex production in control condition (without additions) was set as 100%.

Adding increasing concentrations of crude oils with different percentage compositions in linoleic and oleic acids (see Table 2) confirmed the dose-effect response in stimulating the synthesis of T-A_2-1 _and T-A_2-3 _factors, respectively, and in reducing total T-A_2 _production. Adding oils rich in linoleic acid (*i.e.*, safflower, corn, and cottonseed oils) increased T-A_2-1 _relative productivity up to 30-60% of the total complex, whereas oils rich in oleic acid (*i.e*., olive, almond, canola, and lard oils) enhanced T-A_2-3 _nearly to 30-50% (see representative HPLC profiles in Additional file 2, Figures S4A and S4B). Oils that contain similar amounts of linoleic and oleic acids (*i.e.*, sesame and soybean oils) gave an unusual complex containing comparable amounts of T-A_2-1 _and T-A_2-3 _factors (see HPLC profile in Figure S4C, Additional file 2). The only exception to the common inhibitory action that these additions to T-A_2 _complex production afforded was safflower oil; safflower oil increased the total T-A_2 _titer slightly, but gave a complex composition enriched in T-A_2-1 _and T-A_2-3 _factors, which turned out to be quite different from the control (Table 2).

**Table 2:** Effect of crude oils on teicoplanin production.

		Percentage composition				
		
Addition	Total T-A2	T-A2-1	T-A2-2	T-A2-3	T-A2-4	T-A2-5
None	100	7.3	60.2	13.1	9.1	10.3

Safflower oil (80% L. - 11% O.)						
2.5 g/L	106	29.0	35.3	21.0	4.7	10
5 g/L	126	20.5	41.7	22.1	5.1	10.6
10 g/L	98	24.8	37	22.3	5.3	10.6

Corn oil (60% L. - 25% O.)						
2.5 g/L	111	35.9	23.4	34.0	3.5	3.1
5 g/L	89	40.7	20.4	33.9	2.8	2.2
10 g/L	27	52.9	14.1	26.3	2.5	4.2

Cottonseed oil (58% L. - 14% O.)						
2.5 g/L	90	45.0	24.8	25.7	2.3	2.2
5 g/L	38	54.2	18.7	22.1	3.4	1.6
10 g/L	19	61.4	12.7	13.8	5.2	6.9

Soybean oil (54% L. - 24% O.)						
2.5 g/L	91	24.6	34.6	31.4	4.8	4.6
5 g/L	94	38.2	22.0	33	4.0	2.8
10 g/L	33	45.7	16.1	25.9	4.5	7.8

Sesame oil (38% L. - 53% O.)						
2.5 g/L	66	21.3	29.7	38.9	5.2	4.9
5 g/L	53	32	19.1	40.2	5.8	2.9
10 g/L	27	38.2	13.7	41	5.2	1.9

Olive oil (8% L. - 78% O.)						
2.5 g/L	57	7.2	44.3	34.1	8.3	6.1
5 g/L	49	5.6	46.4	33.7	8.3	6.0
10 g/L	21	8.5	26.9	47.1	13.9	3.6

Almond oil (20% L. - 75% O.)						
2.5 g/L	48	12.7	44.6	31.1	6.2	5.4
5 g/L	46	11.8	43.1	30.4	8.2	6.5
10 g/L	19	15.4	29.3	37.3	13.5	4.5

Canola oil (23% L. - 60% O.)						
2.5 g/L	47	9.7	48.9	26.8	8.3	6.3
5 g/L	47	13.8	41.3	34.1	6.2	4.6
10 g/L	23	16.2	27.6	38.7	13.5	4.0

Lard oil (10% L. - 46% O.)						
2.5 g/L	55	8.5	53.8	21.1	9.5	7.1
5 g/L	30	12.5	39.8	28.8	14.0	4.9
10 g/L	35	16	28.1	42.5	9.7	3.7

*A. teichomyceticus *ATCC 31121 was grown in TM1 medium added at the time of inoculation with different oils. Sample were harvested at 144 hours and analyzed by HPLC as reported in the Methods. Total T-A_2 _complex production in control condition (without additions) was set as 100%. Oil composition in linoleate (L.) and oleate (O.) percentages was reported from [29].

Factors T-A_2-2_, T-A_2-4_, and T-A_2-5 _are characterized by branched fatty acids: 8-methylnonanoic (iso-C10:0), 8-methyldecanoic (anteiso-C11:0), and 9-methyldecanoic (iso-C11:0) acid, respectively (Figure 1). Their precursors were identified as the cell membrane fatty acids 14-methyl pentadecanoic (iso-C16:0), 14-methylesadecanoic (anteiso-C17:0), and 13-methyltetradecanoic (iso-15:0) acid, whose biosynthesis was significantly enhanced by adding amino acids L-valine, L-isoleucine, and L-leucine to the medium [7,18-20]. Isobutyrate, isovalerate, and 2 methylbutyrate represent the starting molecules for synthesizing even-carbon iso acids, odd-carbon iso acids, and odd-carbon anteiso acids, but they could not be directly added to *A. teichomyceticus *cultures owing to their toxicity; thus they were indirectly supplied by adding the precursor amino acids, as shown in Table 1. L-valine increased the production of T-A_2-2 _to 70-80% of the T-A_2 _complex, whereas L-isoleucine or L-leucine increased the relative yields of T-A_2-4 _to more than 30% or T-A_2-5 _to more than 20%, respectively. Increasing concentrations of L-leucine and L-isoleucine progressively inhibited T-A_2 _complex composition, whereas the addition of L-valine caused a marked improvement (60-90% more) in total T-A_2 _productivity.

**Figure 5:**
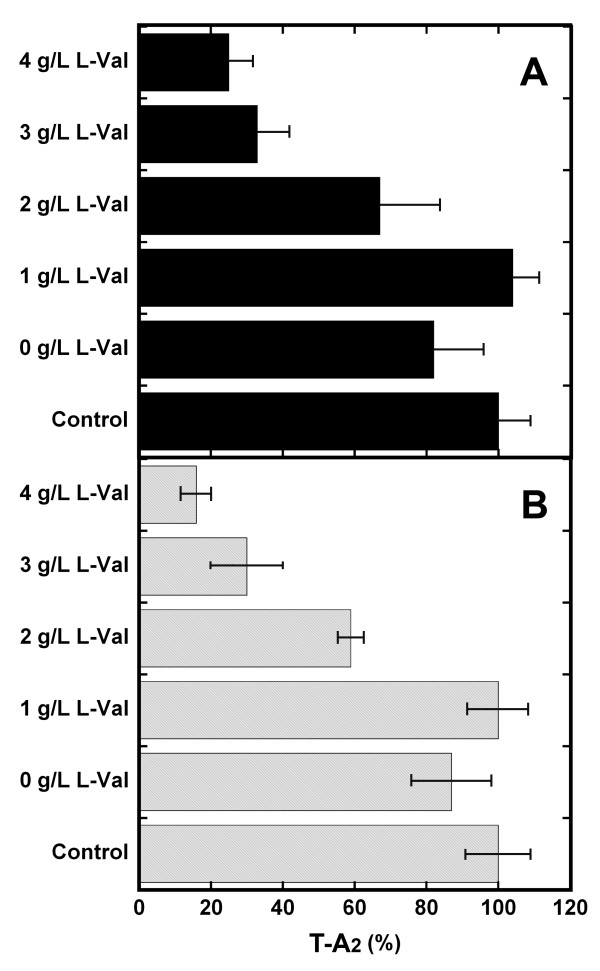
**Production of teicoplanin in TM1 to which L-valine and crude oils were added**. Fermentation of *A. teichomyceticus *ATCC 31121 was run in TM1 to which 2.5 g/L corn oil (**A**) or 2.5 g/L olive oil was added (**B**) and different concentrations of L-valine. T-A_2 _production in control conditions (without oil and L-valine addition) was set as 100%.

The positive effect of L-valine on total T-A_2 _productivity was combined with the addition of corn oil and olive oil, which, when used alone, stimulated T-A_2-1 _and T-A_2-3 _production but reduced total antibiotic productivity (Table 2). Figure 5 shows that the combined addition of 2.5 g/L of oils and of 1 g/L of L-valine restored T-A_2 _productivity to the control level, counteracting the inhibitory effect of adding oil alone. These results indicate that complex composition can be modulated by maintaining the specific effects exerted by biosynthetic precursors, without affecting total antibiotic productivity. Figures S5 and S6 in the Additiona file 2 show the HPLC profiles representing the combined effect of L-valine and selected oils on complex composition.

### Optimized process at fermenter scale

**Figure 6:**
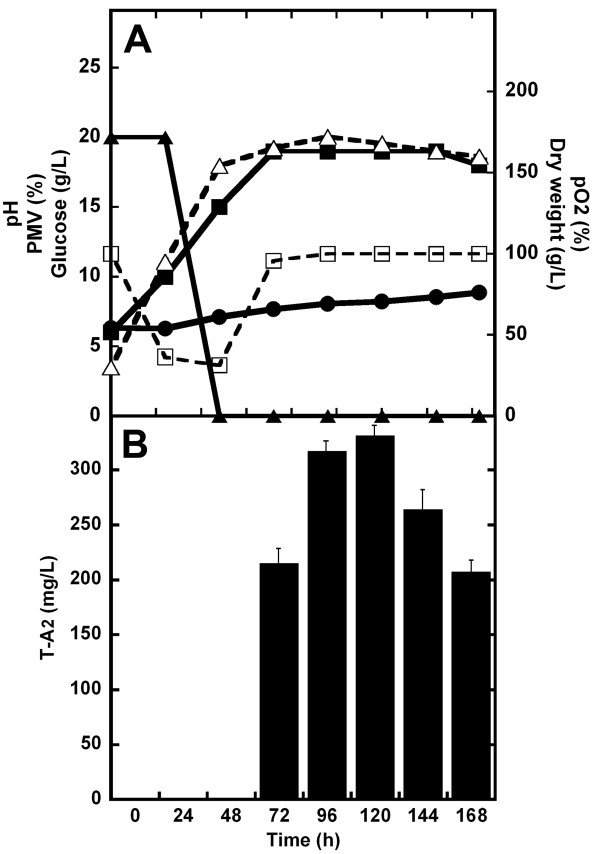
**Growth and teicoplanin production in 3-L batch fermentations of *A. teichomyceticus *ATCC 31121 in TM1 to which L-valine was added**. L-valine (2 g/L) was added at the time of inoculation. The pH value was naturally self-regulated, whereas the pO_2 _was controlled over the 20% of saturation by adjusting agitation speed. In (**A**), time courses time course of pH (●, solid line), pO_2 _(□, dashed line), glucose (▲, solid line), growth curve measured as dry weight (Δ, dashed line), and PMV (■, solid line). In (**B**), production of T-A_2 _measured by HPLC analysis as mg/L (filled bars).

**Figure 7:**
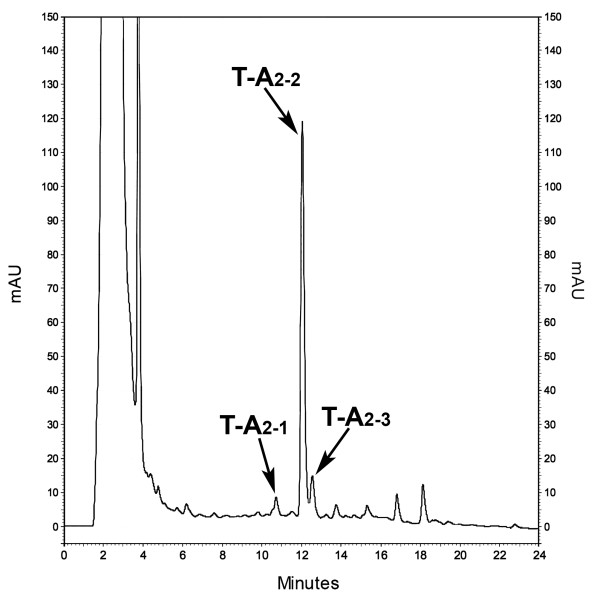
**Teicoplanin profile in TM1 to which L-valine was added**. HPLC profile of sample harvested at 120 hours from the fermentation of *A. teichomyceticus *ATCC 31121 in TM1 to which 2 g/L-valine was added. T-A_2-1_, T-A_2-2_, T-A_2-3_, T-A_2-4_, and T-A_2-5 _represent 7.3, 73.4, 10.5, 2.0, and 6.8% of the total T-A_2_.

The positive effect of adding L-valine to the industrial medium TM1 was confirmed in 3-L fermenters, run as reported above (pO_2 _maintained over the 20% of saturation). As can be seen by comparing Figure 6 with Figure 4, adding amino acid slightly reduced biomass production and prolonged the lag phase (as demonstrated by the reduction in glucose consumption in the first 24 hours). Exponential growth was actually completed in 48 hours and maximum antibiotic productivity was achieved at 96-120 hours. An increase in volumetric (340 mg/L with σ = ± 5 mg/L) and specific (2 mg/g of biomass) productivities of T-A_2 _consititutes the major effect of adding L-valine. As expected, the HPLC profile for T-A_2 _complex composition at 120 hours showed a prevalence of T-A_2-2_, which comprised more than 70% of the complex (Figure 7).

## Discussion

Most of the papers published on teicoplanin production in the last decade [4,11,12,16,21,22] report on efforts to optimize production of industrially valuable mutants, which are produced by applying the classical approach of random mutagenesis and selecting higher producer strains. Classical strain improvement (CSI) still represents the most successful means of meeting the industrial need for a rapid increase in the production yield of antibiotic-producing microbes. Indeed, it generates mutants with randomly uncontrolled, modified genome backgrounds [23]. We recently initiated a program for improving the *A. teichomyceticus *strain based on rational metabolic engineering and recombinant DNA technology, which may provide a novel, alternative strategy for improving titers and that complements the empirical method used in industry. For this purpose we needed to establish a robust and reproducible process with the wild-type strain at different fermentation scales.

Firstly, we developed a complex industrial medium TM1 by single-factor experiments based on the composition of TE/20 medium published in the 1990's [7]; here, we changed the quality and quantity of the essential components such as carbon sources, nitrogen sources, and CaCO_3_. In this complex medium, inorganic phosphate, which is known to inhibit the synthesis of teicoplanin [16] and other teicoplanin-like glycopeptides [24,25] when added to defined mineral media, is slowly released during soybean meal and yeast extract consumption, and its effect has not been further investigated. Since two atoms of chlorine are present in the teicoplanin molecule, we tested the effect of adding different concentrations of NaCl; however, no improvement was observed, suggesting the chlorine content of fermentation medium was already sufficient for producing T-A_2_. Exploiting TM1 medium, the optimal T-A_2 _titer was found to be around 100 mg/L, which is more than five- to ten-fold better than in the initially screened media. An important parameter to be assessed in teicoplanin production -- for the reasons stated in the background section -- is its complex composition, whose factors differ by the length and branching of the fatty acids chains. We were aware that nitrogen sources, and to a lesser extent carbon sources, such as the ones used in the TM1 formulation, contain variable compositions of amino acids and fatty acids. These constituents can supply a pool of precursors for diverse components of the T-A_2 _complex, changing its relative composition in comparison to the ones achieved in other media [7,17].

Secondly, total T-A_2 _production and complex composition in TM1 were found to be reproducible in the miniaturized fermentation system previously described by Duetz [13]. Thus, we were able to handle 24 mini-fermentations in parallel, significantly reducing the workload and the demand for shakers, flasks, media, and analytical equipment during fermentation and strain improvement programs. Previous investigations employing this miniaturized system mostly focused on bacteria with unicellular dispersion and on determining growth and primary metabolism. Only few studies have been published on secondary metabolite production in filamentous streptomycetes, *i.e*., actinorhodin [15] and novobiocin [14]. To our knowledge, this is the first report on antibiotic production in such a miniaturized system by an *Actinoplanes *species, which belong to the so-called rare genera of filamentous actinomycetes [26].

Reproducible total T-A_2 _production and complex composition were also achieved when fermentation was scaled up to laboratory fermenters, where indeed an increase of up to more than 200 mg/L was obtained when DO was kept over 20%. These data confirmed previous observations from a patented high producer mutant at pilot (300 L) and plant (50, 000 L) scales, where it was demonstrated that teicoplanin production decreased significantly as DO was maintained below 20%, indicating that this parameter represents a major concern in scaling up the process [4].

Numerous experts believe that, in a clinical setting, teicoplanin should be treated as an entity, regardless of the relative levels of subcomponents, since their relative amounts, lipophilicity, and MICs do not predict significant differences in pharmacokinetic characteristics. Nevertheless, due to the Pharmacopoeia's specifications with respect to the individual glycopeptide subcomponents (as mentioned in the Background), tools to modulate teicoplanin complexes during manufacturing might be very useful.

To this end, we applied a biosynthetic approach to modulate T-A_2_, which was based on previous investigations of the origin of linear and branched-chain fatty acids constituting the acyl moieties in T-A_2 _[7], in its structurally closely related A40926 lipoglycopeptide [18,20,27], and in the lipopeptide antibiotic ramoplanin [19]. In these antibiotics acyl moieties derive from fatty acid constituents of membrane lipids or from those exogenously added to the growth medium [7,17,20,27]. It was observed that changes in fatty acid composition of cell membranes in the producer actinomycetes caused by feeding precursor amino acids corresponded well to the fatty acid distribution within the produced teicoplanin, A40926, and ramoplanin complexes [7,18-20,27]. Previous studies [18,20] showed that when a single-branched amino acid was added to a minimal medium in *Nonomuraea *sp., which produce the teicoplanin-like A40926, the biosynthesis of membrane fatty acids was influenced not only by the products of the initial steps of its catabolism, but also by the end products of the pathway, depending on the affinity of the starters for the fatty acid synthase. *Nonomuraea *sp.'s fatty acid synthetase preferred isobutyric acid, which is provided by the partial catabolism of L-valine present in the culture medium, whereas isovaleryl-CoA generated from L-leucine was only accepted as a substrate to a lesser extent, and 2-methylbutyryl-CoA from L-isoleucine was not recognized as a promoter. As a consequence of adding L-isoleucine, its complete catabolism provided propionic acid, which was extensively used by the fatty acid synthetase as primer of fatty acids with an odd number of carbons.

A similar interplay between the availability of the biosynthetic starters and the affinity of the starters for the synthase could affect the changes in the membrane composition of *A. teichomyceticus *and consequently the composition of the teicoplanin that is produced. As in the case of A40926 production in *Nonomuraea *sp., L-valine addition improved teicoplanin-specific productivity, suggesting that biosynthesis of the corresponding fatty acid represents a limiting step, which can be overcome by adding the appropriate precursor. The fact that other precursor amino acids and oils stimulated relative production of the corresponding complex components, but inhibited total antibiotic production likely reflects the extent of the membrane perturbation caused by adding massive amounts. In fact, in the cases of both *Nonomuraea *sp. and *A. teichomyceticus*, the major component among the membrane fatty acids, in the absence of added precursors, is the 14-methyl pentadecanoic (iso-C16:0) fatty acid, which originates from L-valine [7,19,20,27]. According to our hypothesis, the increase upon adding L-valine causes a less perturbing effect on membrane composition than adding L-leucine and L-isoleucine, which substantially increases minor fatty acid membrane components, or oils containing long chain fatty acids that can alter membrane properties [19]. Restoration of control productivity in the case of combined addition of L-valine and crude oils indirectly confirms the need to maintain a membrane-equilibrated composition.

These findings should stimulate further work to optimize times for adding L-valine and crude oils to the fermentation process of *A. teichomyceticus*, in order to obtain unusual complex compositions without affecting membrane stability in the early phase of growth. Preliminary data show that the inhibitory effect of crude oils on total teicoplanin production is lower if they are added when the growth rate tends to be decreasing and the cells are entering into the stationary phase of growth. Taking into account the good performance achieved at the fermenter level in TM1 to which L-valine was added (T-A_2 _volumetric productivity more than 300 mg/L), further experiments will be run in oil-fed batch fermentations.

## Conclusions

Most of the cost and quality of teicoplanin depends on fermentation conditions, which typically change when scaling up to an industrial process using valuable high producer strains. We have developed a robust fermentation process by using an industrial medium, which can be scaled down and up and in which complex composition can be modulated by the combined addition of suitable precursors such as amino acids and crude oils. This work was carried out in the wild-type strain ATCC 31121, which has a clear genetic background. This is important for starting a rational improvement program and also helps to better control teicoplanin production during process and strain development.

## Methods

### Microrganism

*A. teichomyceticus *ATCC 31121 was maintained as a frozen vegetative stock at -80°C in 15% v/v glycerol at a biomass concentration of approximately 0.08 g/mL DW (dry weight). This working cell bank (WCB) was prepared by streaking lyophilized mycelium on slants of SM agar medium [28]. After growth, the mycelium from the slant was homogenized in 10 mL of physiological solution (0.9% w/v NaCl) and inoculated into 50 mL liquid E25 [18] in a 300-mL baffled Erlenmeyer flask. Flasks were incubated for 72 hours at 28°C and 220 rpm. Mycelium was collected by centrifugation, washed by water, weighted, resuspended in 15% v/v glycerol and stored as a WCB in 1.5 mL cryo-vials at -80°C.

### Cultivation procedures

To start the fermentation process, one vial of the WCB was inoculated into 300-mL baffled flasks containing 50 mL of vegetative medium E25. Flask cultures were incubated for 72 hours on a rotary shaker at 220 rpm and 28°C and then used to inoculate (5% v/v): (*i) *24-Square Deep Well polypropylene microtiter plates (24-SDW MTPs) purchased from EnzyScreen BV (Biopartner center, Leiden, The Netherlands) containing 2.5 mL production medium; *(ii) *300-mL baffled Erlenmeyer flasks containing 50 mL production medium, or *(iii) *3 L P-100 Applikon glass reactor (height 25 cm, diameter 13 cm) equipped with a AD1030 Biocontroller and AD1032 motor, containing 2 L production medium. Microtiter plates and flasks were incubated at 28°C and 220 rpm for 144-168 hours. Cultivations in fermenters were carried out for 144-168 hours at 30°C, with stirring at 500-900 rpm (corresponding to 1.17-2.10 m/s of tip speed) and 2 L/min aeration rate. Dissolved oxygen (measured as % pO_2_) was monitored using an Ingold polarographic oxygen electrode and controlled by setting agitation speed in cascade with a set point of 20% of saturation with pO_2_. The pH values of culture broths were monitored using a pH meter and controlled by adding H_2_SO_4 _(2, 5% v/v). Foam production was controlled by adding Hodag antifoam through an antifoam sensor. Fermentation runs were replicated at least three times for each experimental condition. Data were calculated as mean values from three replicated fermentations.

### Production media

E1 [10], TE/20 [7], Medium 1 [11], and Medium 2 [12] were tested as production medium. Components of TE/20 medium (carbon sources, nitrogen sources, and CaCO_3_) were examined individually to determine their contribution toward the production of T-A_2 _in *A. teichomyceticus *ATCC 31121 (single-factor experiments).

Different concentrations of L-valine, L-isoleucine, L-leucine, methyl linoleate, methyl oleate, trilinoleate, trioleate, almond oil, canola oil, corn oil, cottonseed oil, lard oil, safflower oil, sesame oil, and soybean oil, previously sterilized in an autoclave were added at the time of inoculation to determine their effect on teicoplanin production. All the amino acids and the oils used were purchased from Sigma-Aldrich (St. Louis, MO, USA).

### Analyses

Fermentation broth samples were collected at regular time intervals and analyzed. Teicoplanin was extracted by mixing 1 volume of whole culture and 1 volume of borate buffer (100 mM H_3_BO_3 _(Sigma-Aldrich), 100 mM NaOH (Sigma-Aldrich), pH 12). Mixtures were then centrifuged (16, 000 *x g *for 15 min). The supernatant was collected and filtered through a Durapore membrane filter (0.45 μm) (Millipore, Billerica, MA, USA).

The glycopeptide production was estimated by HPLC performed on a 5-μm particle size Symmetry C18 (VWR International LCC, Radnor, PA, USA) column (4.6 × 250 mm) eluted at 1 mL/min flow rate with a 30-minute linear gradient from 15% to 65% of Phase B, followed by 10 minutes with 100% Phase B. Phase A was 32 mM HCOONH_4 _(Sigma-Aldrich) pH 4.5:CH_3_CN (Sigma-Aldrich) 90:10 (v/v) and Phase B was 32 mM HCOONH_4 _pH 4.5:CH_3_CN 30:70 (v/v) mixture. The chromatography was performed with a model 1100 HPLC system (Elite Lachrom VWR Hitachi LLC) and UV detection was at 236 nm. As internal standard, pure samples of teicoplanin (Targocid, Sanofi-Aventis) were used. Three HPLC analyses were repeated on the same sample and data were calculated as mean values of three replicated analyses.

The T-A_2 _concentration in the sample was calculated as follows:

$$\text{T}-{\text{A}}_{2}\;\text{concentration }\left(\text{mg}∕\text{L}\right)=\frac{\text{C }\left(\text{std}\right)\times \text{ A}}{\text{A }\left(\text{std}\right)}\times 2$$

where:

C (std) = concentration (mg/L) of the standard

A = area sum of the peaks T-A_2-1_, T-A_2-2 _, T-A_2-3_, T-A_2-4_, T-A_2-5 _in the sample

A (std) = area sum of the peaks T-A_2-1_, T-A_2-2 _, T-A_2-3_, T-A_2-4_, T-A_2-5 _in the standard

2 = dilution factor

The relative content (%) of each factor of the complex was calculated as follows:

$$\text{T}-{\text{A}}_{2-\text{n}}\left(\%\right)=\frac{{\text{A}}_{\text{n}}\times 100}{\text{A}}$$

where:

A_n _= area of single peak T-A_2-n _in the sample where n = 1, 2, 3, 4, 5

A = area sum of the peaks T-A_2-1_, T-A_2-2 _, T-A_2-3_, T-A_2-4_, T-A_2-5 _in the sample

To estimate growth, mycelium was collected by centrifugation (3, 250 *x g *for 20 min) and washed with 2 mL isotonic saline. Dry weight was measured after 24-hour incubation in a 50°C oven. Alternatively, 10 mL of culture were collected and centrifuged to determine PMV. Glucose was analyzed using the Trinder assay (Sigma Diagnostics, St. Louis, MO, USA).

## Competing interests

The authors declare that they have no competing interests.

## Authors' contributions

FM conceived the project and wrote the paper. CT and LG performed medium optimization experiments, developed the HPLC method, and co-wrote the paper. LF carried out fermentation experiments at fermenter scale. GLM prepared *A. teichomyceticus *inoculum, performed miniaturized experiments, analyzed the data, and prepared the figures and the tables. All authors have read and approved the final manuscript.

## Supplementary Material

Additional file 1 **Effect of different conditions of DO and pH on growth and T-A_2 _production**. **Figure S1 **shows that T-A_2 _titer decreased when 300-ml Erlenmeyer flasks were filled with increasing working volumes of the production medium TM1. **Figure S2 and S3 **show growth and T-A_2 _production time courses at 3-L batch fermentations under different conditions of pH and DO controls.
**Figure S1 - Effect of fermentation working volume on growth and T-A_2 _production at flask scale**. Growth followed as PMV (●, solid line) and T-A_2 _production (empty bars) by *A. teichomyceticus *ATCC 31121 were measured in 300-ml Erlenmeyer flasks filled with increasing working volumes of production medium. T-A_2 _production in standard protocols (300-ml Erlenmeyer flasks filled with 50 ml medium) was set as 100%.
**Figure S2 - Growth and teicoplanin production in 3-L batch fermentations of *A. teichomyceticus *ATCC 31121**. In this run, the pH value and the pO_2 _were naturally self-regulated. In (**A)**, time courses pH (●, solid line), pO_2 _(□, dashed line), glucose (▲, solid line), and growth curve measured as PMV (■, solid line). In (**B**), production of T-A_2 _measured by HPLC analysis as mg/L (filled bars).
**Figure S3 - Growth and teicoplanin production in DO- and pH-controlled 3-L batch fermentations of *A. teichomyceticus *ATCC 31121**. In this run, the pH value controlled by adding acid after 48 hours from inoculation and pO_2 _was controlled over the 20% of saturation by adjusting agitation speed. In (**A)**, time course of pH (●, solid line), pO_2 _(□, dashed line), glucose (▲, solid line), and growth curve measured as PMV (■, solid line). In (**B)**, production of T-A_2 _measured by HPLC analysis as mg/L (filled bars). Click here for file

Additional file 2 **HPLC profiles of samples from *A. teichomyceticus *fermentations in TM1 to which crude oils were added**. **Figures S4 A, B and C **show representative HPLC profiles of samples from *A. teichomyceticus *fermentations in TM1 to which crude oils were added. **Figure S5 **shows a comparison of HPLC profiles in the case of addition of corn oil and L-valine. **Figure S6 **shows a comparison of HPLC profiles in the case of addition of olive oil and L-valine.
**Figure S4 - HPLC profiles of teicoplanin production in TM1 to which crude oils were added**. Samples from *A. teichomyceticus *ATCC 31121 fermentation in TM1 to which 2.5 g/L corn oil (**A**), olive oil (**B**) and sesame oil (**C**) were added and collected after 144 hours after inoculation.
**Figure S5 - Comparison of HPLC profiles of teicoplanin production in TM1 to which corn oil and L-valine were added**. Samples from *A. teichomyceticus *ATCC 31121 fermentation in TM1 without any addition (solid line), 2.5 g/L corn oil added (dotted line), and 2.5 g/L corn oil and 1 g/L L-valine added (solid line).
**Figure S6 - Comparison of HPLC profiles of teicoplanin production in TM1 to which olive oil and L-valine were added**. Samples from *A. teichomyceticus *ATCC 31121 fermentation in TM1 without any addition (solid line), 2.5 g/L olive oil added (dotted line), and 2.5 g/L olive oil and 1 g/L L-valine added (dashed line). Click here for file

## References

[B1] Jovetic S Zhu Y Marcone GL Marinelli F Tramper J β-Lactam and glycopeptide antibiotics: first and last line of defense? Trends Biotechnol 2010 28 12 596 604 10.1016/j.tibtech.2010.09.004 20970210

[B2] Zhanel GG Calic D Schweizer F Zelenitsky S Adam H Lagace-Wiens PR Rubinstein E Gin AS Hoban DJ Karlowsky JA New lipoglycopeptides: a comparative review of dalbavancin, oritavancin and telavancin Drugs 2010 70 7 859 886 10.2165/11534440-000000000-00000 20426497

[B3] Rossolini GM Mantengoli E Montagnani F Pollini S Epidemiology and clinical relevance of microbial resistance determinants versus anti-Gram-positive agents Curr Opinion Microbiol 2010 13 5 582 588 10.1016/j.mib.2010.08.006 20846900

[B4] Jung HM Jeya M Kim SY Moon HJ Kumar Singh R Zhang YW Lee JK Biosynthesis, biotechnological production, and application of teicoplanin: current state and perspectives Appl Microbiol Biotechnol 2009 84 3 417 428 10.1007/s00253-009-2107-4 19609520

[B5] Sosio M Kloosterman H Bianchi A de Vreugd P Dijkhuizen L Donadio S Organization of the teicoplanin gene cluster in Actinoplanes teichomyceticus Microbiology 2004 150 Pt 1 95 102 14702401 10.1099/mic.0.26507-0

[B6] Li TL Huang F Haydock SF Mironenko T Leadlay PF Spencer JB Biosynthetic gene cluster of the glycopeptide antibiotic teicoplanin: characterization of two glycosyltransferases and the key acyltransferase Chem Biol 2004 11 1 107 119 15113000 10.1016/j.chembiol.2004.01.001

[B7] Borghi A Edwards D Zerilli LF Lancini GC Factors affecting the normal and branched-chain acyl moieties of teicoplanin components produced by Actinoplanes teichomyceticus J Gen Microbiol 1991 137 3 587 592 1827835 10.1099/00221287-137-3-587

[B8] Malabarba A Strazzolini P Depaoli A Landi M Berti M Cavalleri B Teicoplanin, antibiotics from Actinoplanes teichomyceticus nov. sp. VI. Chemical degradation: physico-chemical and biological properties of acid hydrolysis products J Antibiot (Tokyo) 1984 37 9 988 999 10.7164/antibiotics.37.988 6238927

[B9] EDQM Monografia della Farmacopea Europea 2006 18 Pharmaeuropa Pharmaeuropa

[B10] Coronelli C Beretta G Bardone MR Parenti F Antibiotic substances US Patent 4,239,751 1980

[B11] Jin ZH Wang MR Cen PL Production of teicoplanin by valine analogue-resistant mutant strains of Actinoplanes teichomyceticus Appl Microbiol Biotechnol 2002 58 1 63 66 10.1007/s00253-001-0872-9 11833531

[B12] Lee JC Park HR Park DJ Son KH Yoon KH Kim YB Kim CJ Production of teicoplanin by a mutant of Actinoplanes teichomyceticus Biotechnol Lett 2003 25 7 537 540 10.1023/A:1022842203917 12882141

[B13] Duetz WA Microtiter plates as mini-bioreactors: miniaturization of fermentation methods Trends Microbiol 2007 15 10 469 475 10.1016/j.tim.2007.09.004 17920276

[B14] Siebenberg S Bapat PM Lantz AE Gust B Heide L Reducing the variability of antibiotic production in Streptomyces by cultivation in 24-square deepwell plates J Biosci Bioeng 2009 109 3 230 234 20159569 10.1016/j.jbiosc.2009.08.479

[B15] Minas W Bailey JE Duetz W Streptomycetes in micro-cultures: growth, production of secondary metabolites, and storage and retrieval in the 96-well format Antonie Van Leeuwenhoek 2000 78 3-4 297 305 11386352 10.1023/a:1010254013352

[B16] Heydorn A Suhr-Jessen T Nielsen J Growth and production kinetics of a teicoplanin producing strain of Actinoplanes teichomyceticus J Antibiot (Tokyo) 1999 52 1 40 44 10.7164/antibiotics.52.40 10092196

[B17] Lazzarini A Borghi A Zerilli LF Ferrari P Colombo L Lancini GC Novel teicoplanins by directed biosynthesis J Antibiot (Tokyo) 1997 50 2 180 183 10.7164/antibiotics.50.180 9099232

[B18] Beltrametti F Jovetic S Feroggio M Gastaldo L Selva E Marinelli F Valine influences production and complex composition of glycopeptide antibiotic A40926 in fermentations of Nonomuraea sp. ATCC 39727 J Antibiot (Tokyo) 2004 57 1 37 44 10.7164/antibiotics.57.37 15032484

[B19] Brunati M Bava A Marinelli F Lancini G Influence of leucine and valine on ramoplanin production by Actinoplanes sp. ATCC 33076 J Antibiot (Tokyo) 2005 58 7 473 478 10.1038/ja.2005.63 16161487

[B20] Jovetic S Feroggio M Marinelli F Lancini G Factors influencing cell fatty acid composition and A40926 antibiotic complex production in Nonomuraea sp. ATCC 39727 J Ind Microbiol Biotechnol 2008 35 10 1131 1138 10.1007/s10295-008-0392-z 18651189

[B21] Song JM Park JT Lee HS Kang JH Kang DJ Production of teicoplanin from Actinoplanes teichomyceticus ID9303 by adding proline Biosci Biotechnol Biochem 2008 72 6 1635 1637 10.1271/bbb.80143 18540082

[B22] Park HR Lee JC Hwang JH Park DJ Kim CJ Glycerol affects the acyl moieties of teicoplanin components produced by Actinoplanes teichomyceticus MSl2210 Microbiol Res 2009 164 5 588 592 10.1016/j.micres.2007.05.007 17659865

[B23] Chen Y Smanski MJ Shen B Improvement of secondary metabolite production in Streptomyces by manipulating pathway regulation Appl Microbiol Biotechnol 86 1 19 25 10.1007/s00253-009-2428-3 PMC3472513 20091304

[B24] Gunnarsson N Bruheim P Nielsen J Production of the glycopeptide antibiotic A40926 by Nonomuraea sp. ATCC 39727: influence of medium composition in batch fermentation J Ind Microbiol Biotechnol 2003 30 3 150 156 12687487 10.1007/s10295-003-0024-6

[B25] Technikova-Dobrova Z Damiano F Tredici SM Vigliotta G di Summa R Palese L Abbrescia A Labonia N Gnoni GV Alifano P Design of mineral medium for growth of Actinomadura sp. ATCC 39727, producer of the glycopeptide A40926: effects of calcium ions and nitrogen sources Appl Microbiol Biotechnol 2004 65 6 671 677 10.1007/s00253-004-1626-2 15138731

[B26] Lazzarini A Cavaletti L Toppo G Marinelli F Rare genera of actinomycetes as potential producers of new antibiotics Antonie Van Leeuwenhoek 2001 79 3-4 399 405 11816986

[B27] Zerilli LF Edwards DM Borghi A Gallo GG Selva E Denaro M Lancini GC Determination of the acyl moieties of the antibiotic complex A40926 and their relation with the membrane lipids of the producer strain Rapid Commun Mass Spectrom 1992 6 2 109 114 10.1002/rcm.1290060208 1504339

[B28] Beltrametti F Consolandi A Carrano L Bagatin F Rossi R Leoni L Zennaro E Selva E Marinelli F Resistance to glycopeptide antibiotics in the teicoplanin producer is mediated by van gene homologue expression directing the synthesis of a modified cell wall peptidoglycan Antimicrob Agents Chemother 2007 51 4 1135 1141 10.1128/AAC.01071-06 17220405 PMC1855507

[B29] Mittelbach M Remschmidt C Biodiesel: the comprehensive handbook 2006 Graz, Austria: Karl-Franzens University

