# Supplementary figures and images for: Actinoplanes teichomyceticus ATCC 31121 as a cell factory for producing teicoplanin
Source: Microb Cell Fact. 2011 Oct 18;10:82. doi: 10.1186/1475-2859-10-82 (PMC3250949; doi:10.1186/1475-2859-10-82)

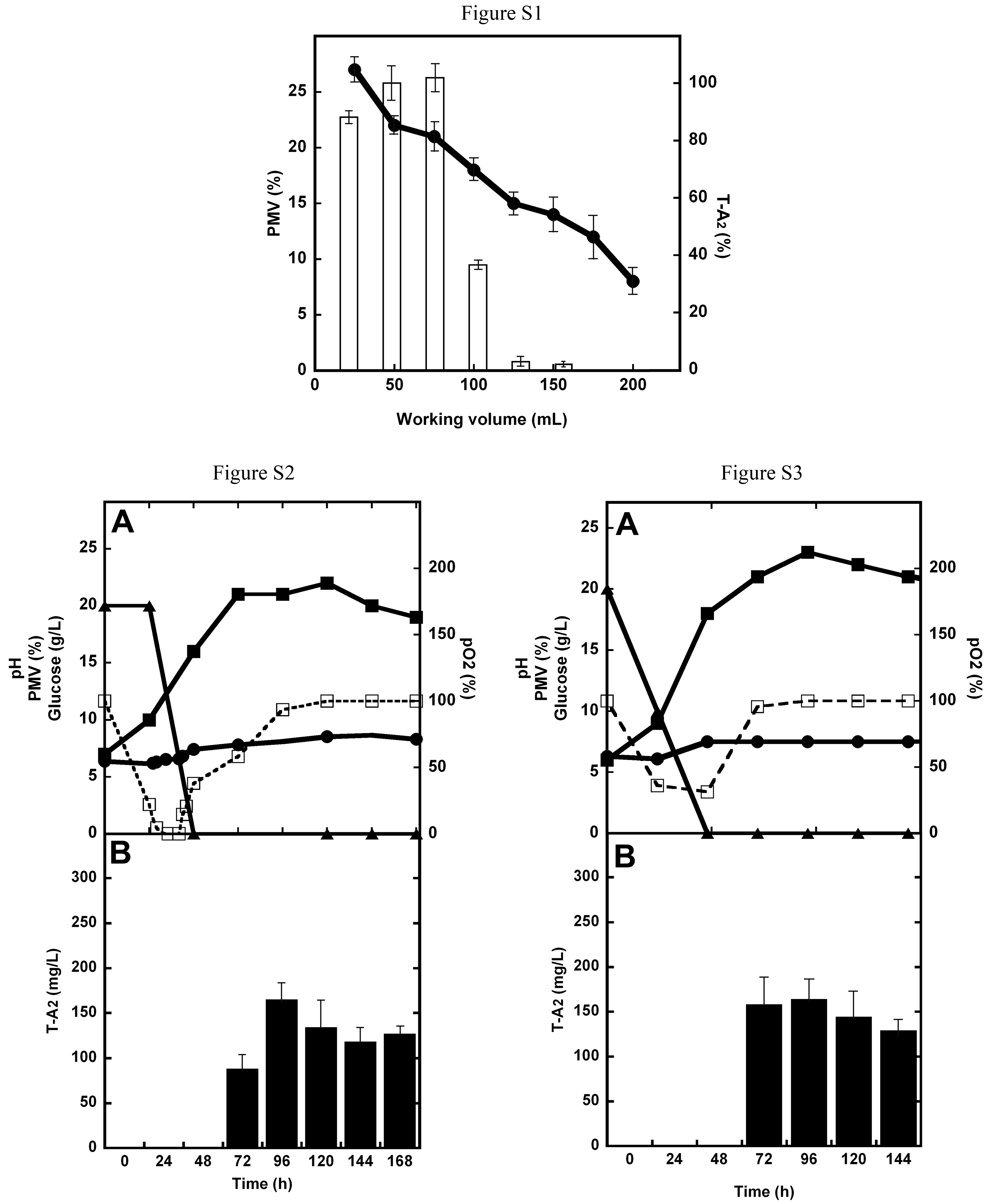

Supplement: Additional file 1 — Effect of different conditions of DO and pH on growth and T-A2 production. Figure S1 shows that T-A2 titer decreased when 300-ml Erlenmeyer flasks were filled with increasing working volumes of the production medium TM1. Figure S2 and S3 show growth and T-A2 production time courses at 3-L batch fermentations under different conditions of pH and DO controls. Figure S1 - Effect of fermentation working volume on growth and T-A2 production at flask scale. Growth followed as PMV (●, solid line) and T-A2 production (empty bars) by A. teichomyceticus ATCC 31121 were measured in 300-ml Erlenmeyer flasks filled with increasing working volumes of production medium. T-A2 production in standard protocols (300-ml Erlenmeyer flasks filled with 50 ml medium) was set as 100%. Figure S2 - Growth and teicoplanin production in 3-L batch fermentations of A. teichomyceticus ATCC 31121. In this run, the pH value and the pO2 were naturally self-regulated. In (A), time courses pH (●, solid line), pO2 (□, dashed line), glucose (▲, solid line), and growth curve measured as PMV (■, solid line). In (B), production of T-A2 measured by HPLC analysis as mg/L (filled bars). Figure S3 - Growth and teicoplanin production in DO- and pH-controlled 3-L batch fermentations of A. teichomyceticus ATCC 31121. In this run, the pH value controlled by adding acid after 48 hours from inoculation and pO2 was controlled over the 20% of saturation by adjusting agitation speed. In (A), time course of pH (●, solid line), pO2 (□, dashed line), glucose (▲, solid line), and growth curve measured as PMV (■, solid line). In (B), production of T-A2 measured by HPLC analysis as mg/L (filled bars). [file 1475-2859-10-82-S1.TIFF]

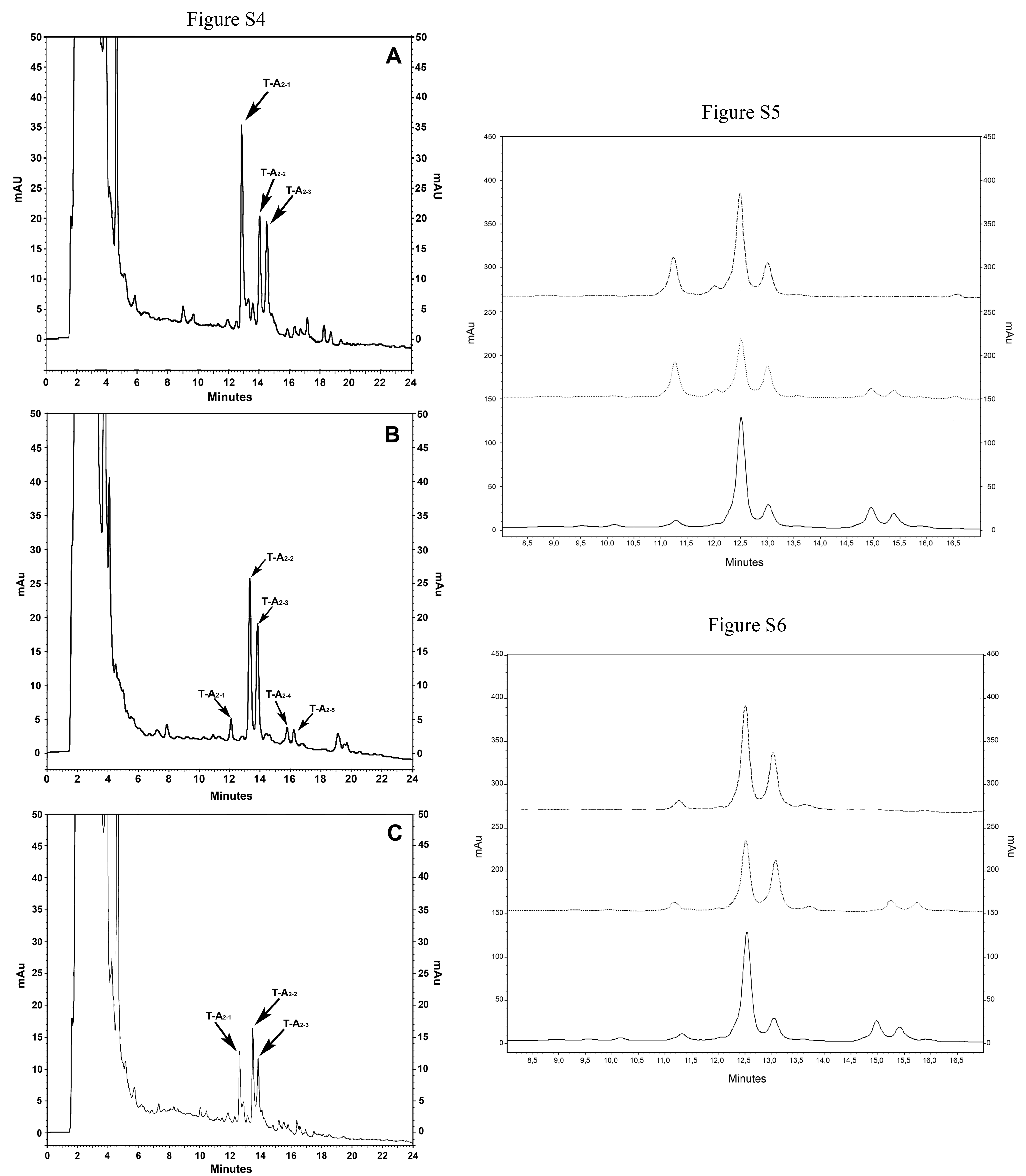

Supplement: Additional file 2 — HPLC profiles of samples from A. teichomyceticus fermentations in TM1 to which crude oils were added. Figures S4 A, B and C show representative HPLC profiles of samples from A. teichomyceticus fermentations in TM1 to which crude oils were added. Figure S5 shows a comparison of HPLC profiles in the case of addition of corn oil and L-valine. Figure S6 shows a comparison of HPLC profiles in the case of addition of olive oil and L-valine. Figure S4 - HPLC profiles of teicoplanin production in TM1 to which crude oils were added. Samples from A. teichomyceticus ATCC 31121 fermentation in TM1 to which 2.5 g/L corn oil (A), olive oil (B) and sesame oil (C) were added and collected after 144 hours after inoculation. Figure S5 - Comparison of HPLC profiles of teicoplanin production in TM1 to which corn oil and L-valine were added. Samples from A. teichomyceticus ATCC 31121 fermentation in TM1 without any addition (solid line), 2.5 g/L corn oil added (dotted line), and 2.5 g/L corn oil and 1 g/L L-valine added (solid line). Figure S6 - Comparison of HPLC profiles of teicoplanin production in TM1 to which olive oil and L-valine were added. Samples from A. teichomyceticus ATCC 31121 fermentation in TM1 without any addition (solid line), 2.5 g/L olive oil added (dotted line), and 2.5 g/L olive oil and 1 g/L L-valine added (dashed line). [file 1475-2859-10-82-S2.TIFF]
